# Maryland State Dental Association

**Published:** 1892-06

**Authors:** 


AMERICAN JOURNAL

—OF—

DENTAL SCIENCE.

VOL. XXVI. BALTIMORE, JUNE, 1892. NO. 2.

ARTICLE I.—ORIGINAL.

### MARYLAND STATE DENTAL ASSOCIATION.

**The annual session was held May 9 and 10, 1892, in St. James' Hotel, Baltimore; Cyrus M. Gingrich, D.D.S., President, in the chair; W. W. Dunbracco, D.D.S,, Secretary.**

(Continued from page 19.)

### HOW WE TREAT AND FILL THE CANALS OF ABSCESSED TEETH SUCCESSFULLY.

BY WM. A. MILLS, D. D. S.

Of all the ills to which human flesh is heir, one of the most painful, and the most difficult to be combated by the dental practitioner, is an abscessed tooth.

How often have the most sanguine seen their fondest hopes decay, when those whom they had dismissed as cured return like Hamlet's ghost to haunt them, after they had reared some elaborate golden superstructure above a smouldering volcano, the presence of which they little dreamed.

It has been customary in treating these cases to have the patient visit the office every other day for two or three weeks for medication before filling.

Much valuable time has been lost to the busy practitioner, who is ofttimes compelled to leave more remumerative and pleasant work to attend those who called for treatment.

Besides, we have been using medicines requiring delicate and cautious handling, the foul odors of which filled our offices and clothing, reminding the uninitiated very forcibly of the smells emanating from a soap-boiling or a fish-fertilizer factory.

Nothing in our practice for years gave us more worry than attempting to treat abscessed teeth; for uncertainty seemed to hang over us from the beginning to the end.

In our opinion, our failures were not caused by not following the course of treatment, laid down in text books, and by authorities in the profession, but were because the treatment was not the correct one to follow in all cases.

We are thoroughly confirmed in this opinion, for we have seen failures from the hands of those more skillful than our own.

Again and again have we been tempted to launch out into the deep waters of total extraction in all cases of abscessed teeth,for fear of the uncertainties of the hidden rock, upon which the reputation of many practitioners has been wrecked.

But being conscious we owed a duty to our patients to try at least to save their teeth, whether abscessed or not, we continued on, feeling our way through the darkness by what light we had, hoping, perchance, we might in the course of time find the harbor of success.

We can never forget the advice once given by an old practitioner to a young man, who had just begun practice. Said he, "Young man, if you wish to make a reputation and want to keep it, never, I say, never fill under any conditions abscessed teeth. Pull them out. If you fill them, nine times out of ten you will fail to make a cure, and nine times out of ten, your patient will seek the advice of some other fellow; and the other fellow will call you a 'quack' for having tried to do your duty. He will make a fee, and you will lose your reputation and a patient. No, sir, young man, if teeth are abscessed, sooner than take any risk, pull them out every time."

This was rather hard on abscessed teeth, and harder still on the fellow who lost both patient and reputation. Who of us has not had a similar experience? for *we* more than once, in our earlier practice, felt the power of the unprincipled man who had neither respect for code of ethics or conscience, but only lived to prey upon the reputations of his professional brothers.

Some three years ago, after considerable experimenting with different remedial agents, we adopted a new system of treatment, the sheet anchor of which is listerine.

Since that time, so great has been our success that all fear of any evil consequences has passed away in the treatment of abscessed teeth, under any conditions, save where the tooth has not sufficient alveolus to support it.

To-day we have greater confidence in it than ever, as it has proven such a haven of rest from the many evils of the old system.

Excuse us, if we seem too sanguine upon the subject.

If we have had failures, we know nothing of them, but this we do know that with a few exceptions, we have kept trace of all cases treated, and our success has been beyond our most sanguine expectations.*

Our system is as follows: After opening the canal, or canals, in the tooth to be treated, as is customary in the

---

*Since writing the above, two cases have been brought to our notice which were failures—one was a case of endostosis (exostosis), the other was the deception on the part of the patient in not treating the tooth until three days before coming to the office. Result: tooth saved after faithful treatment.

treatment of abscessed teeth, we prescribe the following to be applied by the patient:

Listerine Pure (St. Louis Ph'l Co) - ℥ iii

Sig. Saturate a pledget of cotton, and plug it loosely in the pulp chamber, and with another pledget rolled rather hard, force this in on the first pledget, and plug tight. Do this *night* and *morning* for two or three weeks, and let no food enter the cavity at any time.

If any swelling or soreness should follow within 24 hours, instruct patient to pack cotton loosely for two or three days.

The object of tightly plugging the cavity is to force the liquid through the canal to the seat of the disease. We try to impress upon the mind of the patient that successful treatment is not with us alone, but more on the part of the faithful carrying out of our instructions, and keeping the tooth thoroughly medicated; otherwise all our labor will come to nought.

If the abscess is an acute one, before prescribing the listerine, we give the following:

R

Tinct. Calendula - - - -
Aqua, - - - - - - aa ℥ ii
*M*

Sig. Bathe the gums freely and frequently.

After all soreness and swelling has subsided, the patient is given the listerine treatment.

One of the greatest advantages of listerine is that it is cleanly and non-poisonous, and can be used almost as freely as water.

When patient returns for final treatment, before proceeding to fill, we pass a nerve broach into the canal, but not through it, for fear of wounding the parts beyond the apex and creating a blood-clot. If on the withdrawal of the broach, we find a strong odor of listerine, we proceed to fill; but if we find the *slightest* trace of foulness, we dismiss the patient to continue treatment for another week, with the hint that the instructions given have not been faithfully performed.

Before filling the canal, or canals, we use the following:

R Iodoformum Pulv. - ℥ ss.
Creosote Lignum, add q. s. ft. paste.

Sig. Place a small quantity of the paste in the opening of the canal, following it up with stiff oxy-phosphate of zinc, and filling the canals clear to the apex of the tooth.

In all cases, after filling and finishing the operation, paint the gums surrounding the tooth with tinct. iodine.

DISCUSSION OF DR. MILLS' REPORT.

Dr. W. S. Twilley.—I have experimented with this treatment considerably. The idea of listerine was suggested to me by Dr. Mills. I have found it very successful.

Dr. W. A. Mills.—Dr. Volck says that he has been very successful with it, except in one or two cases.

Dr. H. B. Noble.—It seems to me that if the patient put in the cotton that he would get more saliva than listerine in the roots of the tooth.

Dr. E. P. Keech.—Dr. Mills failed to go into the pathology of the abscess at all. He refers to a case which he had treated in this way. It had proved a failure and he found that the cause of the failure was because exostosis had taken place. But I never knew that it could take place upon a dead tooth. I have been practicing for quite a long time, and I am quite sure that exostosis cannot take place upon a tooth which has lost its vitality.

Now in regard to listerine. It is an antiseptic. It is a most valuable preparation for the mouth. But as far as the treatment of an abscess is concerned, there are many other materials that seem better adapted for this purpose. It is difficult to pass such things up the canal of the tooth, difficult to get them up to the apex. Sometimes after working for an hour, we do not succeed in getting the fluid clear to the apex of the tooth. Hence I argue that if the patient is allowed to apply the cotton saturated with listerine, the result will be a failure in nine cases out of ten. I would say to these young men that if you propose to experiment with the treatment, don't experiment with a tooth that you value."

Dr. W. S. Twilley.—I must confess that I differ from Dr. Keech. I have used listerine and one reason why I use it, is because it is so harmless and does such good work. I instruct the patient in applying the listerine to keep the tooth dry and having saturated a piece of cotton with the fluid to force it in with another pellet of cotton rolled hard. I know that the success that I have had in treating teeth in this manner warrants me in continuing with my treatment.

Dr. Cyrus M. Gingrich.—In the treatment of a dead tooth the primary thing is to cleanse the tooth and keep it dry. I think it is of no use to put anything into the tooth until all the dead matter has been removed. After the canal has been thoroughly cleansed and is then kept dry, I believe we have accomplished about all that can be done for it. Then we believe that the cavity must be hermetically sealed.

Dr. Mills.—When I read my paper I said: "After opening the canal and treating it in the usual manner." *Always* the first thing to do is to thoroughly clean it and remove all the dead matter. Gentlemen, I say to you, take some case and try it, and if it fails, then come out and report.

Dr. B. Myer.—In the treatment of an abscessed tooth you need to force the medicine through the abscess until it comes out on the outside. The tooth must not be filled until the abscess is burned out and new flesh has taken its place.

### HEMORRHAGE AFTER EXTRACTION.

BY CHAS. C. HARRIS, M. D., D. D. S., CHAIRMAN EXECUTIVE COMMITTEE.

*Gentlemen :*

Fearing that some committee would fail to make a report, the President, Secretary and myself at a late hour decided to write a small article each and combine them as a substitute therefor.

We all extract teeth, and occasionally serious trouble arises from post-hemorrhage. We are too often the assisting cause of this annoyance through the practice of a *useless* and *thoughtless* system or habit of giving the patient a glass of water with which to wash out the mouth. This is surely unnecessary and if we stop to think, we are doing what should be carefully avoided; we are encouraging hemorrhage. What we aim at is the cessation of bleeding, and this is accomplished by the blood clotting. Now is it not plain that water washes away the blood—not permitting it to clot ? Experience shows that the first clot is less easily disintegrated than secondary clots. It may be that the wound at first is healthy while later serum oozes from the surface and thus prevents the accumulating corpuscles from taking hold, or the surface having had sufficient time to take on inflammation the blood will coagulate with difficulty.

In case there is a number of teeth to extract or we should wish to be rid of the blood in our effort to reach roots of teeth then give the patient a glass of water *as hot as can be borne.* Hot water encourages blood-setting, and is universally used by surgeons during their operations. In case of obstinate hemorrhage, what is to be done? Anything and everything in preference to those much used and exceedingly objectionable agents—sub-sulphate of iron and per-sulphate of iron. So far as I know these are the most objectionable of all styptics to be used in the mouth. Why? Because the clot formed by this agent is so readily disintegrated and will nearly always come away with "recurring hemorrhage." Recurring or secondary hemorrhage will almost universally occur when dealing with a smooth surface. The reason why it does not always recur when sub-sulphate is used by the dentist is no admirable quality of the agent itself, but because it is used in conjunction with a simple pellet of cotton. It is likely this cotton and not the sub-sulphate of iron that has given the desired result. The cotton would act far better alone. Sub-sulphate of iron is an exceedingly irritating medicine and always leaves an inflamed surface. No wound can heal by "first adhesion" after its use. I think the objectionable features of the sulphates of iron very likely due to the irritating influence of the sulphuric acid used in their preparation. Here let me say that the acid exists in sufficient strength to act deleteriously on tooth structure. Surgeons use it only where an inflammatory result is desired —where sloughing is sought—where adhesions are cut asunder—for example, where an operation is performed on the womb for contracted os. The dentist may use it where the fraenum is cut or adhesions of the cheek to the gums are severed. What must we use? A *non-irritating* astringent, one which experience proves a permanent styptic, one not likely to create inflammation, least likely followed with secondary hemorrhage most of all one that experience has proven.

Alum is one of the most efficient, chloride of zinc is very desirable and also nitrate of silver than which no agent is surer. I approve of using them in the order named. With these agents we need fear no after hemorrhage of the mouth, unless it be one of those rare cases we have read about in which the whole mouth surface "floods" from a wound in one part. In this case a plaster impression must prove most efficacious.

What if we are without a styptic—without so much as a pellet of cotton? Then use nature's own and greatest agent, the finger. Put your finger (or better the patient's finger) over the tooth socket, and the bleeding ceases. Hold it securely in position and the blood cannot flow, the clot will become hard and effective.

I will cite a case. Mr. S. had a bicuspid tooth extracted about three p. m. Being a plethoric subject and a victim of malaria for many years and at this time incapacitated for work because of the frequency of his chills and only able to take exercise walks. The dentist cautioned him to come back in case of renewed hemorrhage. The bleeding was profuse at the time but soon ceased. On getting home it commenced to bleed slightly and after night became alarming, but he thought better not to disturb his dentist and sent to the nearest druggist for something to use. Everybody's agent, the sub-sulphate was sent and used with the most temporary effect. He bled all night and sent for me at 8 a. m. I called, asked him to get up and sit in a chair. This he tried to do but fell over in a faint. We lifted him to the bed. From his appearance and the great quantity of blood in all sorts of utensils I feared he would die. I found his mouth open from a large fibrinous clot, half as big as the fist. I inserted two fingers, withdrew the clot and washed out the socket thoroughly. Dipped a pellet of cotton in some chloride of zinc, then in some nitrate of silver and packed this into the cavity. In five minutes his spittal was as pure and clear as it ever was. I advised a little whiskey every few minutes and all the milk he could drink for a couple days. This was the last I saw of him for a year or more when he informed me that the bleeding had proven a God-send, as he rapidly grew strong and to this time has not had a recurring chill.

Within a couple of days a physician sent me a note by a colored man, stating that the bearer had a tooth extracted a few days before with continued hemorrhage, that he returned to the dentist without relief. Called on him and had been under his treatment a couple of days. He had used internal remedies and the old agent, per-sulphate of iron on a pellet of cotton. Despairing he sent the man to me in so weakened a condition that he could walk with difficulty. I thoroughly washed out the socket, carefully packed a pellet of cotton saturated with chloride of zinc and nitrate of silver. Not another drop of blood was lost. He called the following day to report.

I often stop a bleeding of the gum between the teeth most effectually by springing with *hot* water.

On commencing the above I expected to touch on other topics, but have unwittingly said so much, I close by asking you to discard the sulphates of iron as a styptic.

---

### ADDRESS OF DR. BONWILL.

---

Since dentistry has been associated with medicine, it has begun to make itself allied with kindred sciences. I will show that it has in it a germ of true science when we throw aside our prejudices.

The teeth themselves give us one of the best evidences whether evolution is true or not. This subject has interested me for 34 years. The drawings which I have here have taken me 28 years to produce. The application that I make of the principle that I have found is in fitting artificial teeth.

The equilateral triangle of the lower jaw no other being has, except man and the teeth which are made for that jaw are made according to a natural law that has no gradation. There is no change and there never has undergone any except a retrograde.

Commencing as I did, this work has been simply unfolding nature. Starting from the equilateral triangle and in making these drawings, I have proceeded step by step.

Evolution must be true or untrue. If it is true, then we have the work of an impersonality. But if I can show you that which some other being has designed before me, I have unfolded the work of some other intelligence preceding; a personality to whose intellectual powers our own are allied.

Have the teeth been perpetuated? I had the pleasure of looking at the oldest man known. He was found 20 feet below the surface of the earth. He has been calculated to be over a million years old. I found in his jaw the same equilateral triangle. I have gone over some 4,000 cases individually and each presents the same thing. You find it in the embryo from the time it is taken from the womb.

Dr. Bonwill then went on to show by means of his carefully constructed diagrams how the human teeth in their size and shape all follow a certain definite law; a law which he had gradually traced out, starting with the principle of the equilateral triangle already referred to. He showed that the teeth of the apes, were constructed in accordance with a different law and that there could be no progression or gradation from the teeth of the ape to those of the human jaw. He also showed how perfectly the human teeth and those of all animals were adapted to their purpose and that a perfect jaw will always remain perfect.

In conclusion he said: "Evidently it is intelligence which has so well adapted the teeth to their uses. The teeth are fitted for their places in the jaw just as an architect would fit the blocks of marble for an arch. Do you suppose the teeth would stay in place unless they were properly fitted? Therefore ascribe to some Being the design. Animals also have jaws just as perfect as any being's. Their teeth were made to fit their needs. Look at the individuality of each tooth! Look at the laws of mastication! When I come to consider the different relations of the teeth, how well adapted they are to get the most use out of them, do I feel the least doubt in my mind that they were designed for their purpose by a Being of intelligence? In the relations which the teeth bear to each other in their adaptation to their purpose you will find the same regularity which you give to your own work."

After the completion of his address Dr. Bonwill said that in a short time he would have a book out on the subject which would show in full all his investigations.

DISCUSSION OF DR. BONWILL'S ADDRESS.

Dr. J. B. Rich.—I don't see that we have anything which we can discuss here. As far as I am concerned this matter of development according to these principles is new to me, and I suppose it is to the rest of you. I haven't knowledge enough of the subject to discuss it.

Dr. H. B. Noble.—I am in the same condition as Dr. Rich. The subject is entirely new to me. I did not know that it had been brought down to such exactness. We have a chance to see whether there are not some laws which will make our business more interesting.

When Dr. Bonwill's book is out. I shall be glad to get it and study its principles. In a great many things in dentistry I think that I am only beginning to know what there is to know. I almost wish I were a young man again.

The study of all these laws will bring us not only profit and interest in itself, but all our work will be done with more satisfaction to ourselves.

Dr. Cockerille, of Washington.—Dr. Bonwill has demonstrated to us very clearly never to sacrifice those most important teeth in the mouth, the first molars. In this respect I think he has opened the eyes of most of us.

Dr. Bonwill.—One little thing I would call your attention to—one of the strongest points that the science of dentistry can make against evolution. If nature reproduces herself, from whence come the temporary teeth? At the age of twelve the temporary teeth are all gone. How are they reproduced?

Dr. Darwin cut his own throat. He said, "demonstrate how any organ can be formed except by gradual development and you overthrow my argument." You can answer him with the human teeth.

Dr. Martin.—I have been interested in a similar field. I believe that to Dr. Bonwill belongs the honor of lifting dentistry into the place of a true science. He brings us out a law and says he knows it is a true law. Is not this the way in the whole universe? Look at water, $H_2O$, something put together in different proportions. This occurs in the whole field of chemistry. Everything in chemistry is put together according to fixed and definite laws. This is just what Dr. Bonwill has shown us here. This is the work of mind. Take the bones. Professor F. T. Miles, in speaking of the head of the femur said: "It is built just for its purpose. It is the work of intelligence, like ours but much superior." So we see the human mouth built intelligently but by an intelligence of an infinitely higher order than ours. Dr. Bonwill has brought his branch of the subject of dentistry to a true science as we will find if we follow his work.

*To be continued.*

